# Nanomaterials for Electrochemical Immunosensing

**DOI:** 10.3390/s17051041

**Published:** 2017-05-05

**Authors:** Mingfei Pan, Ying Gu, Yaguang Yun, Min Li, Xincui Jin, Shuo Wang

**Affiliations:** Key Laboratory of Food Nutrition and Safety, Ministry of Education of China, Tianjin University of Science and Technolo, Tianjin 300457, China; panmf2012@tust.edu.cn (M.P.); GuYing0816@126.com (Y.G.); yaguangyun@163.com (Y.Y.); lmlm0919@126.com (M.L.); jin924249256@126.com (X.J.)

**Keywords:** electrochemical immunosensors, metal nanomaterials, carbon-based nanomaterials, semiconductor nanomaterials, review

## Abstract

Electrochemical immunosensors resulting from a combination of the traditional immunoassay approach with modern biosensors and electrochemical analysis constitute a current research hotspot. They exhibit both the high selectivity characteristics of immunoassays and the high sensitivity of electrochemical analysis, along with other merits such as small volume, convenience, low cost, simple preparation, and real-time on-line detection, and have been widely used in the fields of environmental monitoring, medical clinical trials and food analysis. Notably, the rapid development of nanotechnology and the wide application of nanomaterials have provided new opportunities for the development of high-performance electrochemical immunosensors. Various nanomaterials with different properties can effectively solve issues such as the immobilization of biological recognition molecules, enrichment and concentration of trace analytes, and signal detection and amplification to further enhance the stability and sensitivity of the electrochemical immunoassay procedure. This review introduces the working principles and development of electrochemical immunosensors based on different signals, along with new achievements and progress related to electrochemical immunosensors in various fields. The importance of various types of nanomaterials for improving the performance of electrochemical immunosensor is also reviewed to provide a theoretical basis and guidance for the further development and application of nanomaterials in electrochemical immunosensors.

## 1. Introduction

Biosensors are a special kind of devices with specific recognition ability, which usually employ specific biological macromolecules such as enzymes, receptors, or antibodies as recognition elements [1]. Owing to their specific recognition or rapid catalysis of the recognition elements, biosensors can meet the requirements of specificity and rapidity, as well as the real-time and on-line detection needs, of the modern analysis process and thus fill a wider research and development space [2]. Immunosensors, an important sub-branch of biosensors, function based on the specific recognition between antigens and antibodies [3,4]. Theoretically, all biological macromolecules such as proteins, microbes that can be used as antigens and other compounds, including hormones, antibiotics, persistent environmental pollutants and toxins that can be used as haptens can induce the production of antibodies with specific recognition ability. This feature represents a powerful driving force for the further development of immunosensors, which accordingly represent some of the most in-depth-studied biosensors. In recent years, studies on immunosensors have mainly focused on how to accurately quantify the signal response with respect to the antigen-antibody binding and signal conversion/amplification, which has led to remarkable achievements in the respective fields [5,6,7]. Based on their different forms of signal conversion, immunosensors can be divided into electrochemical, optical, piezoelectric, calorimetric and other forms, among which the electrochemical immunosensors constitute one of the most extensive classes [8,9]. In 2011, the International Union of Pure and Applied Chemistry (IUPAC) defined an electrochemical immunosensor as an integrated device based on an antigen/antibody reaction, which can convert certain chemical substances or their concentration signals into a corresponding electric signal through the sensor element, and realize a specific quantitative or semi-quantitative analysis [10]. With the development and popularization of electrochemical sensing techniques, to date, a variety of electrochemical immunosensors based on different electrochemical signals including current, potential, conductance and impedance have been developed and applied in diverse areas including clinical inspection [11], environmental monitoring [12], and food analysis [13], in which they have played an irreplaceable role.

Nanotechnology is a relatively new high-tech discipline wherein atoms or molecules are manipulated at a nanoscale scale to achieve specific product processing and manufacturing, or their characteristics are utilized to study a specific material. It is worth noting that the development of nanotechnology has opened up a new realm of human understanding of the world [14]. Nanomaterials are a representative material of nanotechnology, that generally refer to a material of which at least one dimension of the three-dimensional (3D) space is within a nano size range (1–100 nm). Nanomaterials have surface effects, small size effects, and macroscopic quantum tunneling effects and exhibit a series of unique mechanical, electrical, optical, magnetic and catalytic properties. Accordingly, they offer broad prospects for development and have been called “the most promising materials” of the 21st century [15]. The rise of nanotechnology and nanomaterials has provided a broader space for the development of bio-analytical chemistry; in particular, biosensors have also become one of the most promising applications for nanomaterials [16,17]. Owing to their special structural features, strong adsorption capacity, reliable orientation performance, biocompatibility and structural compatibility, novel functional nanomaterials can effectively improve the immobilization of biomolecules (such as enzymes, antibody or DNA) [18], label biomolecules [19], catalyze reactions [20,21], promote electron transfer [22], and facilitate electrochemical signal amplification [23], thus providing new approaches for the development and application of bioelectrochemical sensors. In particular, research on nanomaterials has led to remarkable achievements in a variety of electrochemical immunosensors. 

In this review, the working principle, development and new achievements and progress of electrochemical immunosensors based on various types of signal are introduced. The research related to the important role of various kinds of nanomaterials for improving the performance of electrochemical immunosensors is a primary focus of the review, which aims are to summarize the previous studies of nanomaterials and electrochemical immunosensors, promote their coordinated development across diverse research and technology fields, and further expand their practical applications.

## 2. Electrochemical Immunosensors with Different Signals

### 2.1. Amperometric Immunosensors

**Figure 1:**
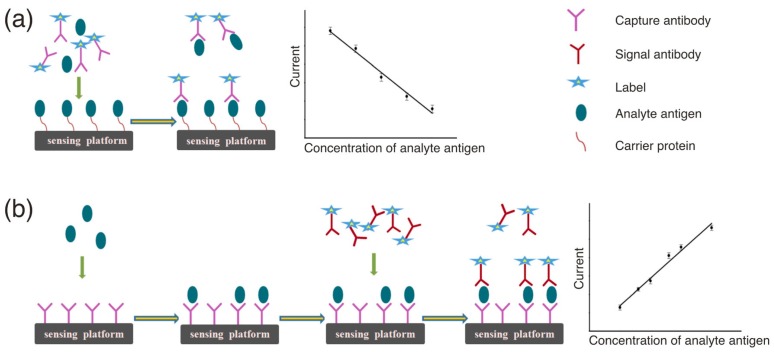
A schematic representation of competitive immunoassays (**a**) and sandwich immunoassays (**b**). Reproduced with permission from reference [39]. Copyright 2016 John Wiley and Sons.

Amperometric immunosensors represent a highly developed biosensing field that has led to the appearance of several commercialized products [24]. This kind of sensors can measure the current change at a constant voltage signifying the occurrence of a redox reaction on the analyte, because the resulting current on the sensing electrode surface is proportional to the analyte concentration [25]. This type of response system is highly sensitive and highly linearly related to the analyte concentration, allowing relatively straight forward data processing and conversion, thus making it ideal for immunochemical sensing. Following the first report from Aizawa et al. [26] in 1979 on an amperometric immunosensor for human chorionic gonadotropin, this type of sensor has attracted much research attention and developed rapidly. Owing to the non-electrochemical activity of the antigen and antibody biomolecules, indirect electrochemical immunoassays are mainly used for measuring the content of immune protein molecules. Specifically, by labeling an electroactive substance or enzyme on an antigen or antibody, the concentration of the antigen or antibody in the sample can be indirectly measured by monitoring the current change caused the reaction of the substrate of the electroactive substance or the enzyme catalyzed reaction between the immobilized biomolecule and the biomolecule in the solution. In this mode, the resulting current signal of the antigen antibody binding reaction has been amplified using the markers such as enzymes (alkaline phosphatase [27,28], horseradish peroxidase (HRP) [29,30] or glucose oxidase [31,32]) or nanomaterials (Au nanoparticles (AuNPs) [33], graphene [34], carbon nanotubes (CNTs) [35,36] or silica nanoparticles [37,38]), which has allowed the detection sensitivity and reproducibility to be increased to a certain extent. Additionally, according to the enzyme-linked immunoassay (ELISA) principle, this kind of immunosensors can be further divided into two categories based on competition or sandwich methodologies (Figure 1) [39].

**Figure 2:**
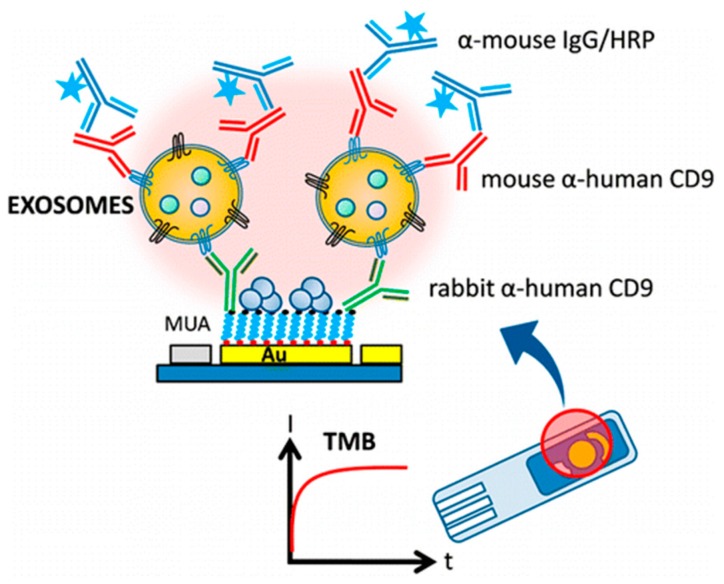
Schematic diagram of amperometric immunosensor using sandwich mode by immobilizing rabbit antihuman CD9 antibodies on gold substrates. Reproduced with permission from reference [40]. Copyright 2016 American Chemical Society.

Doldán et al. [40] fabricated a sandwich mode amperometric immunosensor by immobilizing rabbit antihuman CD9 antibodies on gold substrates and using monoclonal antibodies against CD9 for the detection of captured antigen (Figure 2). Signal amplification is obtained from the fact that multiple detector antibodies bind to the surface of each captured vesicle. This amperometric biosensor can be easily incorporated into future miniaturized and semiautomatic devices for determination. 

A competitive disposable amperometric immunosensor was firstly developed by Manfredi et al. [41] based on gliadin-functionalized carbon/nanogold screen-printed electrodes for rapid determination of celiotoxic prolamins. The developed immunocompetitive assay achieved good sensitivity for gliadin in ethanol extracts, giving limit of detection and limit of quantitation at 8 and 22 ng·mL^−1^, respectively.

### 2.2. Potentiometric Immunosensors

Potentiometric immunosensors comprise a kind of biosensing device that performs immunoassays by measuring the change of electrochemical potential for direct or indirect detection of various antigens with the characteristics of real-time monitoring and rapid response time. In 1975, Janata [42] first reported the change of membrane potential caused by an antigen binding to an antibody immobilized on the surface of a metal electrode using polyvinyl chloride film. When the antibody or antigen was detected by the sensitive membrane of the sensor, the membrane potential (or the electrode potential) could be changed, which has a logarithmic relationship between the change of potential and antigen or antibody concentration. 

Based on the different principle of membrane-and ion-electrode potential measurement, two kinds of potentiometric immunosensors have been developed. The measurement of membrane potential is susceptible to nonspecific adsorption issues, resulting in a large background current and low detection sensitivity, limiting its further practical application. In contrast, the high sensitivity of an immunoassay is combined with the high selectivity of an ion-selective electrode in the ion selective electrode immunosensor which can monitor the change of electrode potential caused by an antibody-immobilized ionic carrier on the surface of the electrode because of the specific binding of the antigen, further reflecting the concentration of the antigen. For example, an ion-selective immunosensor for the determination of insulin has been constructed by Ghindilis et al [43], in which using lactase labeled antibody for insulin and according that utilizes a lactase-labeled antibody for insulin. According to the catalysis of lactase oxidation, the resulting potential changes on the electrode could be recorded to achieve insulin monitoring. The introduction of nanoparticles provides new means for improving the performance of ion-selective potential immunosensors, especially in terms of increasing the linear response range and enhancing the sensitivity. Fu et al. [44] have adsorbed AuNPs to the surface of the electrode by self-assembly of mercaptoethylamine and then immobilizing the immunoglobulin antibody (anti-IgG) to develop a highly sensitive potentiometric immunosensor. The performance of the AuNPs-introduced sensing device was confirmed by cyclic voltammetry and electrochemical impedance techniques to show a detection limit of 12 ng·mL^−1^, which is similar to that of the ELISA method. Tang et al. [45] have constructed a similar immunosensor on the surface of a glassy carbon electrode using AuNPs for anti-diphtheria immobilization for the detection of diphtheria toxoid in biological samples. The linear response range and detection limit achieved were 2.4–60.0 ng·mL^−1^ and 5.2 ng·mL^−1^, respectively. Notably, these studies also demonstrate that the nanomaterials can effectively solve the shortcomings of low sensitivity and narrow linear range of potentiometric immunosensors. 

### 2.3. Impedance Immunosensor

Electrochemical impedance spectroscopy is an effective method to study the properties of conductive materials and interfaces, and has been widely used in the field of electrochemical sensors [46]. For a sensor with impedance characteristics, the combination of its capacitance, inductance, and resistance characteristics can produce a specific impedance signal. Therefore, when the surrounding environment of the sensor is modified to cause a change of the phenomenon underlies the response of sensors based on electrochemical impedance. Impedance immunosensors can real-time monitor the change on the electron transfer impedance in solutions before and after an immune reaction to achieve assessment of the tested analyte. Compared with other analytical methods, this detection mode is free of the markers, affords less damage to the immune system, and involves relatively simple operation and sensor preparation processes [47].

**Figure 3:**
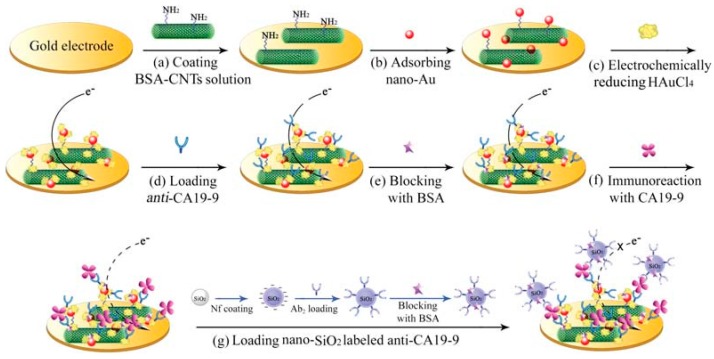
Schematic processes of sandwich impedance immunoassay method using the complex of CNTs and AuNPs as the electrode matrix for antibody immobilization. Reproduced with permission from reference [50]. Copyright 2010 Royal Society of Chemistry.

During the fabrication of impedance sensors, nanomaterials are the most commonly used for antibody immobilization, which not only increases the amount of antibody loaded but also maintains antibody activity over a longer period of time to meet practical needs. For example, Chen et al. [48] reported a simple preparation process of one enzyme-free labeled impedance immunosensor based on 3D-orderedmacroporousgoldfilm. The film was employed to immobilize the antibody for reaction with the corresponding antigen to form immune complexes, which could inhibit the electrical activity of the Fe(CN)_6_^3−/4−^ probe and further achieve the sensitive detection of C-reactive protein. To construct a novel impedance immunosensor, Li et al. [49] prepared a novel ionic-liquid-doped polyaniline inverse opals via electropolymerization method on an electrode surface, wherein AuNPs self-assembly was utilized for antibody immobilization. Yuan et al. [50] developed a sandwich impedance immunoassay using the complex of CNTs and AuNPs as electrode matrix for antibody immobilization. As the capture of silicon nanosphere markers on the electrode surface is not conducive to the transfer of electrons, the electrical activity of the Fe(CN)_6_^3−/4−^ probe was thereby inhibited, achieving the electrochemical determination of the target glycoprotein (Figure 3).

Impedance sensors with advantages of ease of generation, low-cost, and rapid response also represent good prospects in the field of microbial analysis [51]. For example, Yang et al. [52] has constructed a microelectrode impedance sensor without marker to detect *Escherichia coli* O157:H7 in food samples. The target bacteria was adsorbed on the indium tin oxide (ITO) microelectrode surface and caused an impedance response, which had a proportional relationship with the target bacteria concentration in the range of 4.36 × 10^5^–4.36 × 10^8^ cfu/mL. Nevertheless, the detection limit of the impedance sensor is still relatively high compared to that of other methods, and the impedance analysis system for complex samples also needs to be improved, requiring substantial effort to render this technology more practical and popular.

### 2.4. Conductometric Immunosensors

Many chemical reactions are accompanied by the generation and consumption of varieties of plasma that can change the conductivity in solutions, thus allowing the determination of conductivity in a variety of chemical systems. The conductometric immunosensors can detect an antigen by measuring the change of the conductivity caused by the change of the type and number of ions in the solution prior to and following immunization. This measurement is usually carried out by immobilizing a specific enzyme on the metal electrode surface to determine the conductivity under the action of an electric field. Yagiuda et al. [53] has successfully fabricated a novel conductivity-based immunosensor for sensitive detection of methamphetamine in human urine, greatly reducing the detection cost. Muhammad-Tahir et al. [54] reported a conductometric biosensor for detecting foodborne pathogens (*Escherichia coli* O157:H7 and *Salmonella spp*). The developed biosensor consisting of two components: an immunosensor on electrochemical sandwich immunoassay and a reader for signal measurement, provided a specific, sensitive, low volume, and near real-time detection mechanism. 

**Figure 4:**
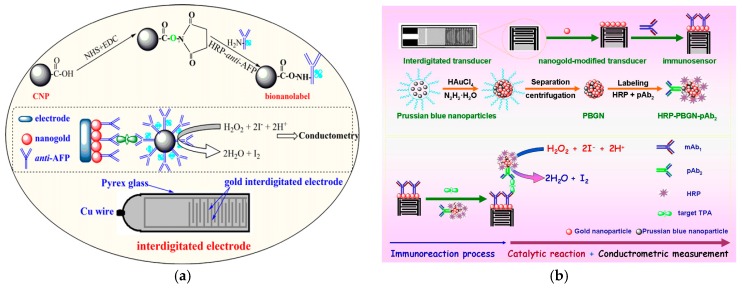
Schematic illustration of the conductometric immunosensor on the interdigitated gold electrode: (**a**) carbon nanoparticles as labels. Reproduced with permission from reference [55]. Copyright 2011 Elsevier; (**b**) biofunctionalized Prussian blue-gold hybrid nanostructure. Reproduced with permission from reference [56]. Copyright 2016 Elsevier.

Tang et al. [55] have designed a simple and sensitive conductometric immunosensor for detection of alpha-fetoprotein (AFP) using carbon nanoparticles as labels. The immunosensing probe was fabricated by means of immobilizing monoclonal AFP primary antibodies on an interdigitated conductometric transducer, while the antibodies for detection were assembled using nanocarbon-conjugated horseradish peroxidase-labeled anti-AFP (CNP-HRP-anti-AFP). A new conductometric immunoassay was also prepared by Xu et al. [56] based on biofunctionalized Prussian blue-gold hybrid nanostructure for sensitive determination of tissue polypeptide antigen (Figure 4).

Although the conductometric immunosensors have been utilized since the early period of immunosensor technologic development, owing to the large influence of ionic strength in the testing samples, buffer volume and the nonspecific adsorption effect in the measurement process, the development of improved detection and analysis using this type of immunosensor has been relatively delayed. 

### 2.5. Capacitive Immunosensors

Electrochemical capacitive immunosensors represent a new type of sensor with remarkable merits of high sensitivity, simple structure, easy integration and no need for labels. In particular, in recent years these have been demonstrated with respect to their capacity to facilitate direct and rapid detection [57]. When the metal electrode is in contact with the electrolyte solution, an electric double layer is generated at the interface between the electrode and the testing solution [58]. The interface capacitance of the testing solution can be sensitive to the physical and chemical properties of the interface changes. Specifically, when the material with low polarity is adsorbed to the electrode surface, the thickness of the interface layer will increase and the interface dielectric constant will be reduced, resulting in the decrease of the interface capacitance. It is well known that the protein molecules represent biomacromolecules with large molecular weight and low polarity. When a protein molecule is adsorbed to the electrode surface, the interface capacitance from the electrode solution is significantly reduced. Thus, in a capacitive immunosensor, the antibody is firstly immobilized on the surface of the electrode, and when the antigen-antibody is bound to the electrode surface, the interface capacitance is correspondingly reduced to reflect the amount of antigen to be detected. For example, Mirsky et al. [59] have compared several methods forthe immobilization of protein molecules on self-assembled monolayers. They determined that by using *N*-hydroxysuccinimide or carbodiimide to activate the carboxyl groups on the ω-mercapto-hexadecanoic acid monolayer, following albumin immobilization on this monolayer, the sensor surface could still maintain high dielectric properties. Accordingly, a detection limit of human serum albumin of 15 × 10^−8^ mol·L^−1^ was achieved using a sandwich capacitive immunosensor.

In general, various types of electrochemical immunosensors have been developed to varying degrees along with the development of various disciplines and new technologies. However, owing to the potential instability, volatility, and other shortcomings of biological identification unit (antibody), the generation of suitable electrochemical immunosensors in terms of stability and reproducibility requires further improvement. The rapid development of biology, informatics, materials science and microelectronics will likely provide a better platform for the development of electrochemical immunosensing technology.

## 3. Electrochemical Immunosensors Based on Various Nanomaterials

In recent years, with the rapid development of nanotechnology, a variety of new nano-scale materials with excellent performance have been developed, providing a wider platform for the construction of high-performance biosensors [60]. The application of nanomaterials in electrochemical immunosensors mainly includes the following aspects [61,62]: (1) as a sensor substrate for the immobilization of biomolecules to increase the loading amount and further enhance the reactivity; and (2) as an antibody/antigen marker without impairing the activity of the biomolecule and the corresponding components. The concentration of the target analyte can be determined based on the electrochemical detection of nanomaterials and the use of nanostructured amplification markers can greatly increase the signal to produce ultra-sensitive electrochemical immunosensors [63]. To date, various nanomaterials including metal-based (Au, Ag, Pt, or Cu nanoparticles or nanoclusters), carbon-based (CNTs, mesoporous carbon, or graphene), semiconductor (SiO_2_ nanoparticles or films, quantum dots (CdTe, PbS or carbon dot), and composite nanomaterials have been applied in the construction of electrochemical immunosensors on different types of signals and have been recognized for their properties of high sensitivity and stability as well as low-cost, thus providing broad application prospects [64,65,66].

### 3.1. Metal Nanoparticles

#### 3.1.1. Au and Ag Nanomaterials

Au metal is a good conductor of electricity and Au materials exhibit strong adsorption and good biocompatibility, which can be applied for the immobilization of biomolecules and to supply a stable environment to effectively maintain enzyme and other biological protein activity. The Au nanomaterials are mainly divided into AuNPs [67,68], Au nanorods [69,70] and Au nanowires [71], among which AuNPs are of the most interesting, owing to their advantages of simple and rapid preparation, high stability, and uniform particle size [72]. Au nanomaterials are usually immobilized on the surface of the electrode by a bifunctional ligation reagent such as *p*-thiophenol [73], 1,6-hexanedithiol [74] or polythionine [75] for physical or chemical adsorption of the capture probes. Antibody recognition molecules can also be linked to AuNPs firstly and subsequently fixed to the surface of the electrodetransducer [76,77].

**Figure 5:**
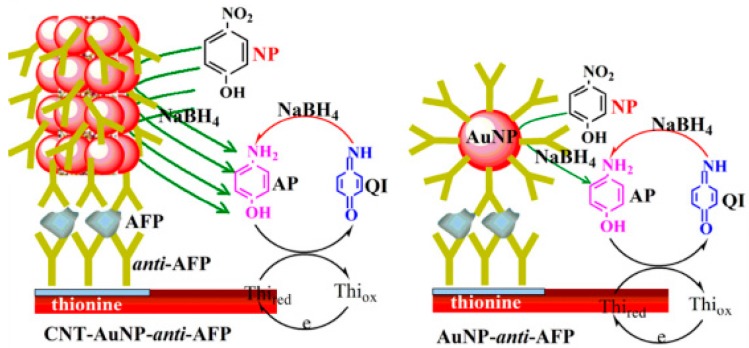
Schematic illustration of sandwich-type electrochemical immunosensor and nanocatalyst-based assay principle: CNT-AuNP-anti-AFP (**left**) and AuNP-anti-AFP (**right**). Reproduced with permission from reference [79]. Copyright 2016 Elsevier.

Das et al. [78] prepared a low-noise immunosensor in an indirect determination mode using an AuNPs labeled secondary antibody to catalyze the reduction of *p*-nitrophenol to electroactive *p*-aminophenol, and the produced electrical activity aids in detecting the antigen concentration. Simultaneously, the sodium borohydride (NaBH_4_) was added into the reaction solution to reduce the reaction product. In a similar approach, Tang et al. [79] used AuNPs to functionalize multi-walled CNTs and employed this new AuNPs-multi-walled CNTs material for antibodies labeling, which were further immobilized on the electrode surface to fabricate an electrochemical immunosensor by sandwich immunoassay. This innovative research also employed the substrate cycling reaction of nitrophenol, NaBH_4_ and thionine catalyzed by AuNPs, and thus amplifying the response current and greatly improving the detection sensitivity. A diagram illustrating the construction of immunosensors and signal amplification principles is shown in Figure 5.

**Figure 6:**
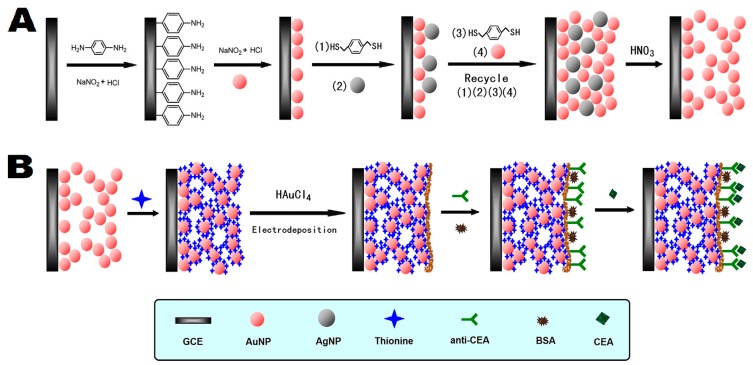
Schematic illustration of GC/Thi@NPG/AuNPs immunosensor (**A**) and competitive immunoreaction (**B**). Reproduced with permission from reference [80]. Copyright 2013 Elsevier.

Furthermore, Sun et al. [80] employed *p*-dimethylmercaptobenzene as a cross-linking agent to obtain a nanoporous gold (NPG) material with high conductivity and large surface area via a layer by layer self-assembly technique involving the alternate assembly of AuNPs and Ag nanoparticles (AgNPs) on the glassy carbon electrode surface, followed by the use of nitric acid to dissolve the AgNPs (Figure 6). The NPG material could absorb a large number of electroactive thionines through the Au-S bond and the electrostatic interaction, further enhancing the sensitivity of the immunosensor with a detection limit achieving to 3 pg·mL^−1^. Chen et al. [81] has reported a type of ordered 3D-Au nanoclusters generated by two-step electrodeposition using the spatial obstruction/direction of the polycarbonate membrane for the fabrication of an amperometric immunosensor. The electrodeposited Au nanoclusters directly formed electrical contacts and immobilization interfaces with protein molecules without post modification and positioning. Bovine serum albumin-picloram was immobilized by self-assembly on the 3D Au nanoclusters and then competitive immunoreaction with the picloram antibody and target picloram was executed and HRP-labeled secondary antibody was applied for enzyme-amplified amperometric measurement. The proposed amperometric immunosensor has exhibited good precision, sensitivity, selectivity, and storage stability.

**Figure 7:**
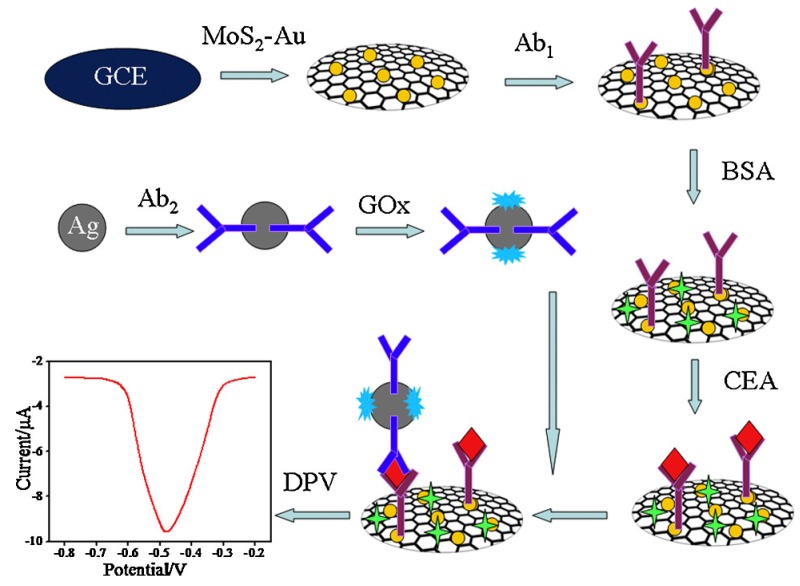
AgNPs-labeled immunosensor based on MoS_2_-Au composite film. Reproduced with permission from reference [85]. Copyright 2015 Elsevier.

Notably, some studies [82,83,84] have reported the further enhancement of AuNPs-labeled molecule biometric signal amplification using other nanomaterials, which provide a secondary amplification of detection signal and improve the detection sensitivity significantly. For example, Wang et al. [85] has reported an AgNPs-labeled immunosensor based on MoS_2_-Au composite film for carcino-embryonic antigen (CEA) detection (Figure 7). 

In this study, a MoS_2_-Au composite film with good catalytic activity toward H_2_O_2_ was firstly applied on a glassy carbon electrode and then the second antibody and glucose oxidase were incoporated modified in AgNPs to obtain an AgNPs-Ab_2_-GO*_x_* composite. The whole detection process was performed using differential pulse voltammetry in H_2_O_2_ solution containing 1% glucose with a linear range of 1 pg·mL^−1^–50 ng·mL^−1^ and a detection limit of 0.27 pg·mL^−1^. Additionally, Chu et al. [86] reported a AgNPs-enhanced colloidal gold-labeled electrochemical immunosensor. The antigen was first immunoreacted with the primary antibody adsorbed onto the polystyrene micropores and then combined with a gold-labeled secondary antibody. After the immune reaction, the AgNPs was added to catalyze the metal reduction to form silver film on the AuNPs. The silver film was then acidolyzed and subjected to electrochemical immunoassay on a glassy carbon electrode by indirect stripping voltammetry. In comparison, Wang et al. [87] synthesized Au-Ag core-shell nanoparticles by an AgNPs-enhanced effect that were used for the detection of platelet-derived growth factor. In the experiment, it was demonstrated that as the increase of the target analyte concentration, more silver metal was deposited on the electrode surface to form a stronger catalytic signal, thus achieving sensitive detection. 

#### 3.1.2. Other Metal Nanomaterials

Nanomaterials of other metals including Cu, Pd, Pt and others have also been incorporated for the fabrication of novel electrochemical immunosensor. Various metal nanomaterials have improved the performance of immunosensors, especially in the aspect of signal amplification to enhance the detection sensitivity [88,89,90]. 

**Figure 8:**
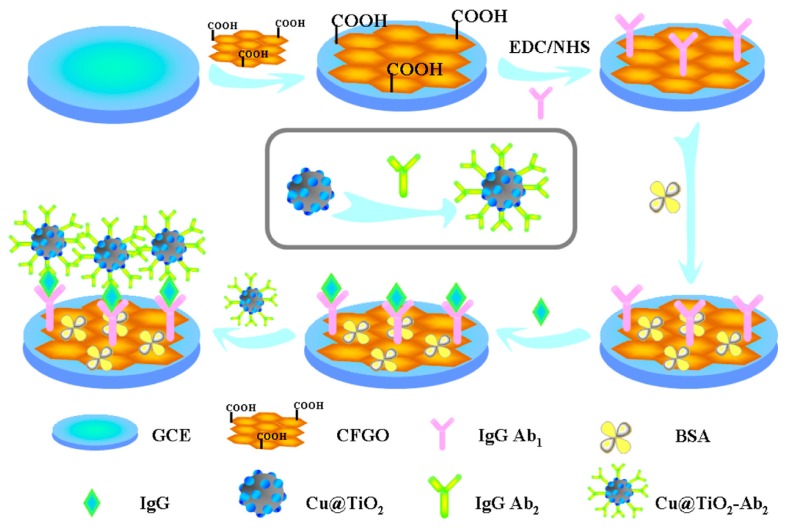
Cu@TiO_2_-labeled sandwich-type electrochemical immunosensors. Reproduced with permission from reference [91]. Copyright 2014 Elsevier.

Zhang et al. [91] have synthesized Cu-doped titanium dioxide nanoparticle (Cu@TiO_2_) and applied it as labels for the fabrication of sandwich-type electrochemical immunosensors on glassy carbon electrode (Figure 8). The prepared Cu@TiO_2_ nanocomposite shows high electrocatalytic activity towards reduction of H_2_O_2_ at the presence of Cu ions. The dual functionality of Cu@TiO_2_ enables the fabrication of immunosensor showing high sensitivity, acceptable stability and good reproducibility for both detection modes (square wave voltammetry: linear range: 0.1 pg·mL^−1^–100 ng·mL^−1^; detection limit: 0.052 pg·mL^−1^; chronoamperometry: linear range: 0.01 pg·mL^−1^–100 ng·mL^−1^; detection limit: 0.0043 pg·mL^−1^).

The different morphologies of metal nanoparticles could affect their properties and suitability for use. For example, Pt nanoparticles with different morphologies such as hollow versus solid Pt nanospheres display different electrochemical characteristics when used as labels in immunosensors and in other applications [92,93]. Cui et al. [94] have developed at a disposable immunosensor array using mesoporous platinum nanoparticles (M-Pt NPs) as nonenzymatic labels. The M-Pt NPs were employed to label the secondary antibody (Ab_2_) for signal amplification in sandwich-type immunoreactions. Using breast cancer related panel of tumor markers (CA125, CA153 and CEA) as model analytes, this proposed immunosensor array showed wide linear ranges of over 4 orders of magnitude with the detection limits of 0.002 U·mL^−1^, 0.001 U·mL^−1^ and 7.0 pg·mL^−1^ for CA125, CA153 and CEA, respectively.

### 3.2. Carbon-Based Nanomaterials

In recent years, carbon-based nanomaterials derived from a range of forms, from C_60_, to CNTs to graphene have become the most active research field in nanoscience, effectively promoting the rapid development of nanotechnology [95,96,97]. Carbon-based nanomaterials usually have good electrical conductivity and biocompatibility, and can improve the active sites of electrochemical reactions [98]. Due to the large specific surface area that increases the amount of immobilized biomolecule, this kind of materials are of interest in the field of electrochemical research.

#### 3.2.1. Carbon Nanotubes (CNTs)

Among one-dimensional nanomaterials CNTs have both a special structure (radius: 2–20 nm, with axial dimensions on the micron scale) and a large specific surface area. The carbon atoms in CNTs form a wide range of delocalized bonds with significant conjugation effects, resulting in substantive conductivity. Based on these excellent features, CNTs have a very wide range of applications in immunosensors [99,100,101,102,103]. Among these, the use of CNTs as electrode modifying substrates or markers to build highly sensitive sandwich-type immunosensors represent an important aspect of their study.

**Figure 9:**
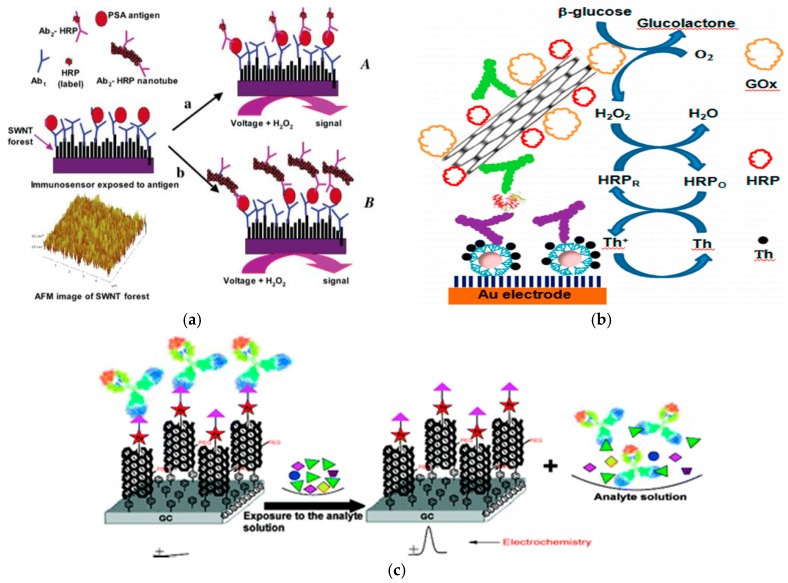
The working principle of this typical CNTs-based immunosensor: (**a**) CNTs-linked HRP for electrode modification. Reproduced with permission from reference [104]. Copyright 2006 American Chemical Society; (**b**) Activated multi-walled CNTs for immobilizing labeled-antibody. Reproduced with permission from reference [105]. Copyright 2013 American Chemical Society; (**c**) Single-walled CNT for immobilizing an antibody. Reproduced with permission from reference [106]. Copyright 2012 American Chemical Society.

Rusling and his colleagues [104] have found that CNTs have good intermolecular electron transfer properties, when they are linked to HRP to modify the electrode. Based on this result, they designed a sandwich electrochemical immunosensor (Figure 9a). Furthermore, a sandwich immunoassay was developed using multi-walled CNTs that included the following steps: (1) CNTs with specific surface area were used to immobilize the primary antibody on the electrode surface; (2) a sandwich reaction was performed to capture enzyme-labeled secondary antibodies loaded on CNTs on the electrode surface; (3) the addition of H_2_O_2_ to the substrate to reflect the concentration of the antigen. Owing to the synergetic effect of HRP and CNTs, the electrochemical response signal of the relative immunosensors is greatly enhanced. The detection limit can reach 4 ng·L^−1^ for the prostate cancer marker PSA and 40 fg·mL^−1^ for 10 μL undiluted bovine serum.

Jeong et al. [105] employed the HRP and glucose oxidase to label an anti-CEA antibody and immobilize the labeled-antibody on multi-walled CNTs activated by dimethylaminopropyl carbodiimide (EDC) and *N*-hydroxysuccinimide (NHS). Use of the multi-walled CNT material significantly increased the immobilized amount of antibody (Figure 9b). This functionalized multi-walled CNT complex was used as a dendritic nanostructure matrix and AuNPs in combination with thionine media to develop a highly sensitive method for the determination of CEA by electrochemical immunoassay. In addition, Liu et al. [106] developed a functional single-walled CNT material for immobilizing antibodies on the surface of a glassy carbon electrode (Figure 9c). The fabricated electrochemical immunosensor could be successfully used for the detection of endosulfan over the range of 0.01–20 ppb with a detection limit of 0.01 ppb in 50 mM phosphate buffer at pH 7.0. Furthermore, Wang et al. [107] employed CNTs as a carrier for covalently bonding a large number of enzyme molecules (approximately 1 μm CNT, representing a load of approximately 9600 enzyme molecules) to prepare an electrochemical immunosensor using CNTs as a marker with a detection limit of 500 fg·mL^−1^ (160 μL in 25 μL sample). Compared with the ordinary enzyme-labeled electrochemical immunosensor, the signal response of this new electrochemical immunosensor can be improved by 100-fold. Using the displacement mode, trinitrobenzene (TNT) was first applied in the SWCNT conduction channel, and then the anti-TNT antibody was attached [108]. When acting with the target or its derivative, the detection of analyte was achieved by monitoring the change of impedance or conductance owing to the displacement reaction. Notably, this novel immunoassay method using the displacement reaction has a relatively wide detection range (0.5–5000 μg·L^−1^ TNT).

#### 3.2.2. Graphene and Graphene Oxide

Graphene exhibits unique physical and chemical properties, in particular a monolithic structure, high conductivity, large specific surface area, no toxicity and good electron mobility, and is widely used in the fields of electrochemical sensing and biosensing [109,110,111,112]. The graphene materials carries a high density of defects on the surface and demonstrates particularly impressive positive electrochemical properties [113]. To date, graphene-modified electrodes have been successfully applied for assessing H_2_O_2_ [114], NADH, dopamine, ascorbic acid, uric acid and acetaminophen. The application of graphene onto a variety of inorganic and organic electroactive materials is further illustrated by its role as a promising new carbon substrate in the field of electrochemical analysis. 

Narayanan et al. [115] strongly modified graphene on the surface of a glassy carbon electrode by a series of covalent reactions, and an antibody was further modified on the graphene using the EDC/NHS cross-linking reagent. Using RaMIgG-ALP/AuNPs as a signal amplification marker, the detection of botulinum neurotoxin E was achieved through sandwich immunization with the detection range and detection limit achieving to 10 pg·mL^−1^–10 ng·mL^−1^ and 5.0 pg·mL^−1^, respectively.

**Figure 10:**
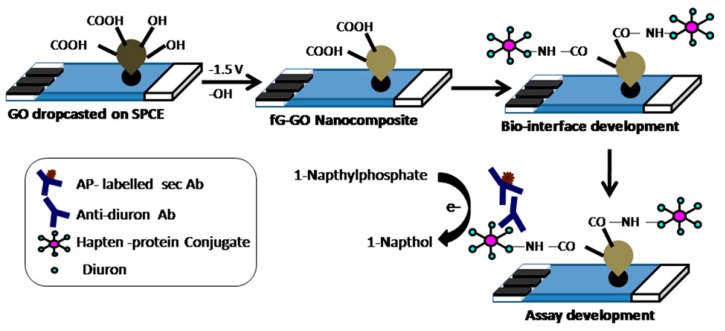
Illustration of in-situ electrochemical synthesis of functionalized graphene-graphene oxide nanocomposite on screen printed electrodes and subsequent immunoassay development. Reproduced with permission from reference [116]. Copyright 2013 Elsevier.

Figure 10 shows an electrochemical method developed by Sharma et al. [116] applied to reduce the graphene on the surface of a screen printed electrode and develop a sandwich electrochemical immunosensor for the detection of diuron. Furthermore, the graphene can also be applied for the construction of immune probes for detection [117]. For example, Li et al. [118] has prepared an Ru-AuNPs/graphene probe for CEA detection. The Ru-AuNPs/graphene probe was synthesized layer by layer using AuNPs, Ru(bpy)_3_^2+^ and poly(dimethyldiallylammonium chloride)-pretreated graphene and further reacted with an antibody immobilized on a nanoporous Au membrane. Yang et al. [119] also fabricated an immune-labeled probe using graphene as a carrier of thionine and HRP to prepare a sandwich electrochemical immunosensor for the detection of tumor markers.

**Figure 11:**
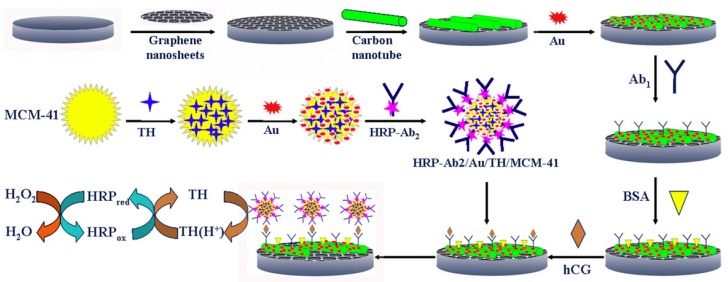
Fabrication process of AuNPs/Thionine/MCM-41 nanomaterials and measurement protocol of the electrochemical immunosensor. Reproduced with permission from reference [124]. Copyright 2012 Elsevier.

With the development of graphene materials, many researchers have introduced or removed some functional groups on their surface to obtain graphene derivatives such as graphene oxide and reduced graphene [120]. Specifically, oxygen-containing functional groups are introduced into the graphene to obtain graphene oxide; subsequently, incomplete removal of these groups by chemical or heat treatment is used to obtain reduced graphene. The resulting graphene derivatives with different properties also have good applications in the field of immunosensors. In particular, Liu et al. [121] designed an immunosensor for the simultaneous detection of CEA and AFP, in which graphene oxide was applied as the platform for immunoreaction. Reduced graphene [122] has also been applied for the fabrication of an immunosensing platform for CEA and AFP. The composites of multi-walled CNTs [123] and graphene provide a 3D electrode interface, which has rich reaction sites for electrochemical reactions, enhancing the electron transfer ability between the active sites of the enzyme and the electrode, and thus improving the detection sensitivity. The AuNPs-dotted CNTs-graphene composite and functionalized mesoporous materials (MCM-41) developed by Lu et al. [124] also significantly enhanced the detection sensitivity of electrochemical immunosensors (Figure 11).

#### 3.2.3. Other Carbon Materials 

Carbon nanofibers have a greater functional surface area and more surface active groups, and thus are considered to be a more promising material [125,126]. Carbon nanospheres have relatively richer functional groups with better biocompatibility, dispersibility, and relative activity [127]. 

Wu et al. [128] first used soluble carbon fiber to fabricate an immunosensor with rapid free isolation. Cui et al. [129] prepared carbon nanospheres using a microwaved hot solution method and further modified the surface of carbon nanospheres with the nano-gold to obtain composite nanometer microspheres with high surface energy and good biocompatibility. The labeled secondary antibody was labeled on the surface of gold-carbon composite nanospheres to obtain highly sensitive electrochemical immunosensors that achieved to a detection limit of 5.6 pg·mL^−1^. In addition, Du et al. [130] used gravimetric and functionalized carbon nanospheres to double the amplification of the electrochemical signal, resulting in a detection signal of the developed immunosensor 7-fold greater than that of normal sensors.

### 3.3. Semiconductor Nanomaterials

Semiconductor nanomaterials primarily including silica nanoparticles and quantum dots, exhibit excellent properties such as large specific surface area, high surface reaction activity, and strong adsorption capacity, providing a new route for biomedical research.

#### 3.3.1. SiO_2_ Nanomaterials

The surface coordination of nano-SiO_2_ particles present with a large number of unsaturated residues and different bonds and hydroxyl groups, allowing this materials to be readily surface-functionalized. As they also exhibit good biocompatibility toward biologically active molecules, nano-SiO_2_ particles represent excellent carriers for the immobilization of proteins and enzymes in electrochemical biosensors [131]. For example, Hong et al. [132] prepared ferrocene-coated SiO_2_ nanoparticles using oil-in-water technology to form stable, homogeneous, biocompatible, and highly surface active interfaces to effectively immobilize the antibodies in a no-reagent electrochemical immunosensor. In particular, this sensor had simple preparation, low cost and high sensitivity characteristics.

**Figure 12:**
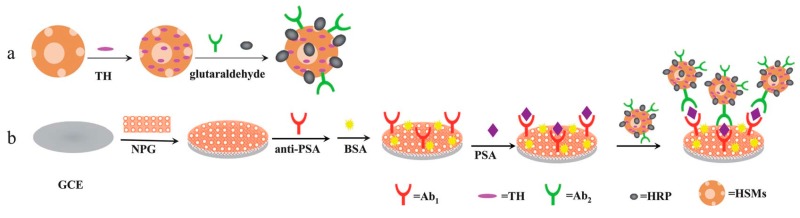
Schematic electrochemical immunosensor based on NPG (**a**) and hollow mesoporous silica microspheres (**b**). Reproduced with permission from reference [133]. Copyright 2011 Royal Society Chemistry.

Figure 12 shows a schematic diagram of a novel electrochemical immunosensor reported by Wu et al. [133] based on NPG and hollow mesoporous silica microspheres (HSMs). Owing to the large specific surface area of amino-functionalized HSMs, the loading amount of antibodies as well as mediators and enzymes thereon was substantially increased, which in turn increased the sensitivity of the immunosensor (limit of detection: 6.00 pg·mL^−1^). This new method may be quite promising with potential broad applications for clinical immunoassays. Additionally, Yang et al. [134] first used a porous silicon ball to adsorb a large amount of thionine, in addition to a large amount of HRP and signal antibodies by covalent loading. Through the catalytic signal amplification from the HRP enzyme, highly sensitive detection of the cancer markers was achieved. Furthermore, the researchers in this group applied a similar method for modifying an ionic liquid onto a porous silicon sphere surface for biomolecules immobilization. Combined with the sandwich immunoassay, an amperometric immunosensor was thereby successfully fabricated for highly sensitive detection of the cancer markers [135].

Tang et al. [136] has added the complex of thionine and HRP enzyme into nano-silica solution, further combining this with an HRP-labeled secondary antibody to construct an electrochemical immunosensor. In this study, the electron mediator and the enzyme label were both modified on the sensor recognition element, simplifying the electrochemical measurement process and resulting in a high detection sensitivity toward the target cancer marker CA125 (limit of detection: 0.1 U·mL^−1^). Wu et al. [137] also prepared a uniform dispersion of silica nanospheres and silane-functionalized surface for labeling signal antibodies and loading high levels of HRP enzyme. This allowed a highly sensitive immunoassay for AFP to be developed in combination with sandwich mode and enzyme-catalyzed cyclic amplification. When the antibody and quantum dots were simultaneously applied onto the surface of the silica nanospheres, this composite signal tracer could greatly improve the electrochemical signal combined with metal ion electrochemical dissolution analysis, achieving highly sensitive detection of the target analytes [138,139].

#### 3.3.2. Quantum Dots

A variety of quantum dots not only have the characteristics of nanomaterials, but also can give good, sharp and sensitive dissolution peaks through electrochemical dissolution voltammetry [140,141,142]. Thus, quantum dot materials have good prospects in the related fields of nanomaterials and electrochemical analysis [143,144,145].

Liu et al. [146] have used different semiconductor nanomaterials (ZnS, CdS, PbS and CuS) for labeling various proteins (α2-microglobulin, IgG, bovine serum albumin, C-reactive protein). According to the peak position of the marker and the tested peak current from the stripping voltammetry, different antigens were identified and determined. 

**Figure 13:**
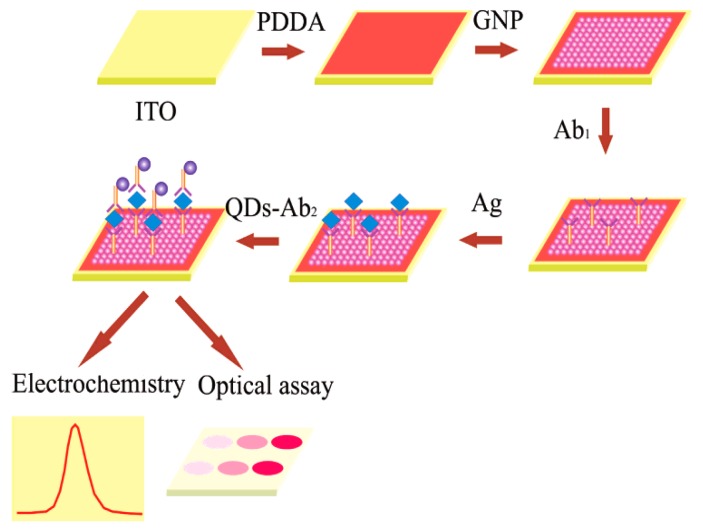
Analytical procedure of fluorescent and electrochemical immunoassay based on CdTe-QDs label. Reproduced with permission from reference [147]. Copyright 2007 American Chemical Society.

Figure 13 shows the analytical procedure of a sandwich-type immunosensor fabricated by Cui et al. [147] on an ITO chip covered with a well-ordered AuNPs monolayer applying CdTe quantum dots as electrochemical and fluorescent labels. The detection sensitivity could be increased to 0.005 ng·mL^−1^ with the linear range of 0.005–100 ng·mL^−1^ by stripping voltammetric analysis. Furthermore, Huang et al. [148] applied a composite film of Au@carbon dots-chitosan in the modification of a glassy carbon electrode for the sensitive and reliable determination of dopamine. The carbon dots contained carboxyl groups with negative charge, which not only provided it with good stability but also enabled the interaction with amine functional groups in dopamine through electrostatic interaction, resulting in multiple-recognition of dopamine with high specificity. 

### 3.4. Other Nanomaterials

Magnetic microspheres represent a new type of magnetic material or nanomaterial developed in recent years. Compared with ordinary magnetic microspheres, magnetic composite microspheres exhibit good magnetic response and nano-synergistic effects in addition to the many characteristics of ordinary microspheres (such as a large surface area), and can also provide more functional groups such as –OH, –NH_2_, –COOH, and –CHO on their surfaces. Some studies have reported the use of magnetic composite nanomaterials in electrochemical immunosensors [149].

Zhou et al. [150] and Tang et al. [151] have reported using magnetic gold nanospheres doped with thionine-labeled HRP and signal antibodies and Prussian blue-labeled HRP-glucose oxidase and signal antibodies to prepare two immunosensors for sensitive detection of CEA. Ding et al. [152] employed streptavid in functionalized magnetic beads to fix a large number of antibodies (Ab1) on the sensor surface for the combination with AuNPs-labeled antibodies (Ab2) and quantum dots. They thus formed a homogeneous sandwich immunoassay to achieve AFP detection through the analysis of stripping voltammetry of quantum dots. A similar immunosensor [153] was also developed for prostate antigen detection by immobilizing Ab1 using carboxylated magnetic nanospheres.

**Figure 14:**
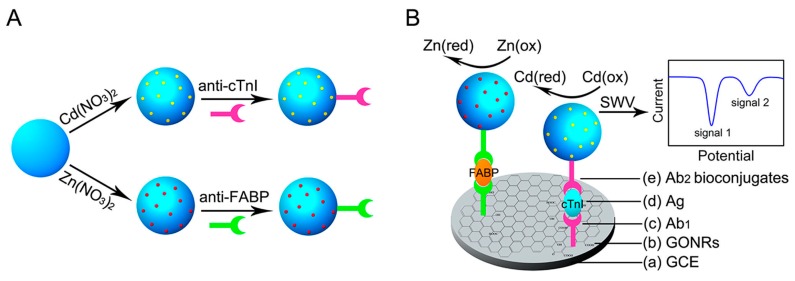
Metal NPs-functionlized phosphopeptide nanospheres as a signal nanoprobe. Reproduced with permission from reference [54]. Copyright 2012 American Chemical Society.

Furthermore, Feng et al. [154] applied metal NPs-functionalized phosphopeptide nanospheres to label Ab2 as a signal nanoprobe (Figure 14). The antibody (Ab1) was immobilized by the oxidized graphene activated by EDC/NHS on the surface of the electrode. The developed sandwich electrochemical immunoassay method can simultaneously determine three proteins: AFP, cardiac troponin I and human myocardial fatty acid binding protein.

## 4. Conclusions

In recent years, various kinds of nanomaterials with different properties have been synthesized, and their applications in electrochemical immunosensor have become increasingly popular, thus leading to a current research hotspot. According to the current status of related research, more in-depth studies are anticipated regarding the following aspects of nanomaterials application in electrochemical immunosensor:

(1) As biomarkers. More electrochemical markers from novel nanofunctional materials will continue to emerge, which will further improve the sensitivity of immunoassays.
(2) As media for different technologies. More new functional nanomaterials with different characteristics will be investigated to meet the mutual promotion and common development requirements of different technologies.
(3) The establishment of nanomaterials for electrochemical online and real-time immune-analysis in the living body remains challenging. Thus, the development of new bio-sensing chips based on nanomaterials is expected.

It is believed that with the development of science and technology and the expansion of related research, a greater number of new nanomaterials with high performance will be applied to electrochemical immunosensors, thus providing broad prospects for their development.
